# Artificial intelligence applied to magnetic resonance imaging reliably detects the presence, but not the location, of meniscus tears: a systematic review and meta-analysis

**DOI:** 10.1007/s00330-024-10625-7

**Published:** 2024-02-22

**Authors:** Yi Zhao, Andrew Coppola, Urvi Karamchandani, Dimitri Amiras, Chinmay M. Gupte

**Affiliations:** 1 Imperial College London School of Medicine, Exhibition Rd, South Kensington, London, SW7 2BU UK; 2 Imperial College London NHS Trust, London, UK

**Keywords:** Artificial intelligence, Deep learning, Magnetic resonance imaging, Meniscus tear, Diagnosis

## Abstract

**Objectives:**

To review and compare the accuracy of convolutional neural networks (CNN) for the diagnosis of meniscal tears in the current literature and analyze the decision-making processes utilized by these CNN algorithms.

**Materials and methods:**

PubMed, MEDLINE, EMBASE, and Cochrane databases up to December 2022 were searched in accordance with the Preferred Reporting Items for Systematic Reviews and Meta-analysis (PRISMA) statement. Risk of analysis was used for all identified articles. Predictive performance values, including sensitivity and specificity, were extracted for quantitative analysis. The meta-analysis was divided between AI prediction models identifying the presence of meniscus tears and the location of meniscus tears.

**Results:**

Eleven articles were included in the final review, with a total of 13,467 patients and 57,551 images. Heterogeneity was statistically significantly large for the sensitivity of the tear identification analysis (*I*^2^ = 79%). A higher level of accuracy was observed in identifying the presence of a meniscal tear over locating tears in specific regions of the meniscus (AUC, 0.939 vs 0.905). Pooled sensitivity and specificity were 0.87 (95% confidence interval (CI) 0.80–0.91) and 0.89 (95% CI 0.83–0.93) for meniscus tear identification and 0.88 (95% CI 0.82–0.91) and 0.84 (95% CI 0.81–0.85) for locating the tears.

**Conclusions:**

AI prediction models achieved favorable performance in the diagnosis, but not location, of meniscus tears. Further studies on the clinical utilities of deep learning should include standardized reporting, external validation, and full reports of the predictive performances of these models, with a view to localizing tears more accurately.

**Clinical relevance statement:**

Meniscus tears are hard to diagnose in the knee magnetic resonance images. AI prediction models may play an important role in improving the diagnostic accuracy of clinicians and radiologists.

**Key Points:**

*•* *Artificial intelligence (AI) provides great potential in improving the diagnosis of meniscus tears.*

*•* *The pooled diagnostic performance for artificial intelligence (AI) in identifying meniscus tears was better (sensitivity 87%, specificity 89%) than locating the tears (sensitivity 88%, specificity 84%).*

*•* *AI is good at confirming the diagnosis of meniscus tears, but future work is required to guide the management of the disease.*

**Supplementary Information:**

The online version contains supplementary material available at 10.1007/s00330-024-10625-7.

## Introduction

The accurate and timely diagnosis of meniscus tears is crucial for effective patient management and optimized treatment outcomes. Magnetic resonance imaging (MRI) has emerged as a valuable diagnostic tool, providing high-resolution images for assessing meniscal pathologies. However, interpreting MRI scans requires expertise and is subject to inter-observer variability [1]. In recent years, artificial intelligence (AI) has gained significant attention as a promising solution for improving diagnostic accuracy and efficiency in orthopedics [2].

While existing literature has highlighted good sensitivity (78–92%) and specificity (88–95%) with MRI diagnosis, radiologists’ experience, scan sequences, and image quality were poorly reported, thereby raising concerns about the applicability of these results in centers with less experience in MRI interpretation [3, 4]. White et al showed that the inter-observer agreement among ten radiologists was low (0.49–0.77). Given the increasing demand for MRI scans in knee injuries, the expertise required in reporting knee MRI scans, and the shortage of highly trained MSK radiologists in many countries, centers often have long waiting times for reports to be generated [5–7]. Therefore, strategies are required to improve the quality and timing of MRI reports.

**Fig. 1:**
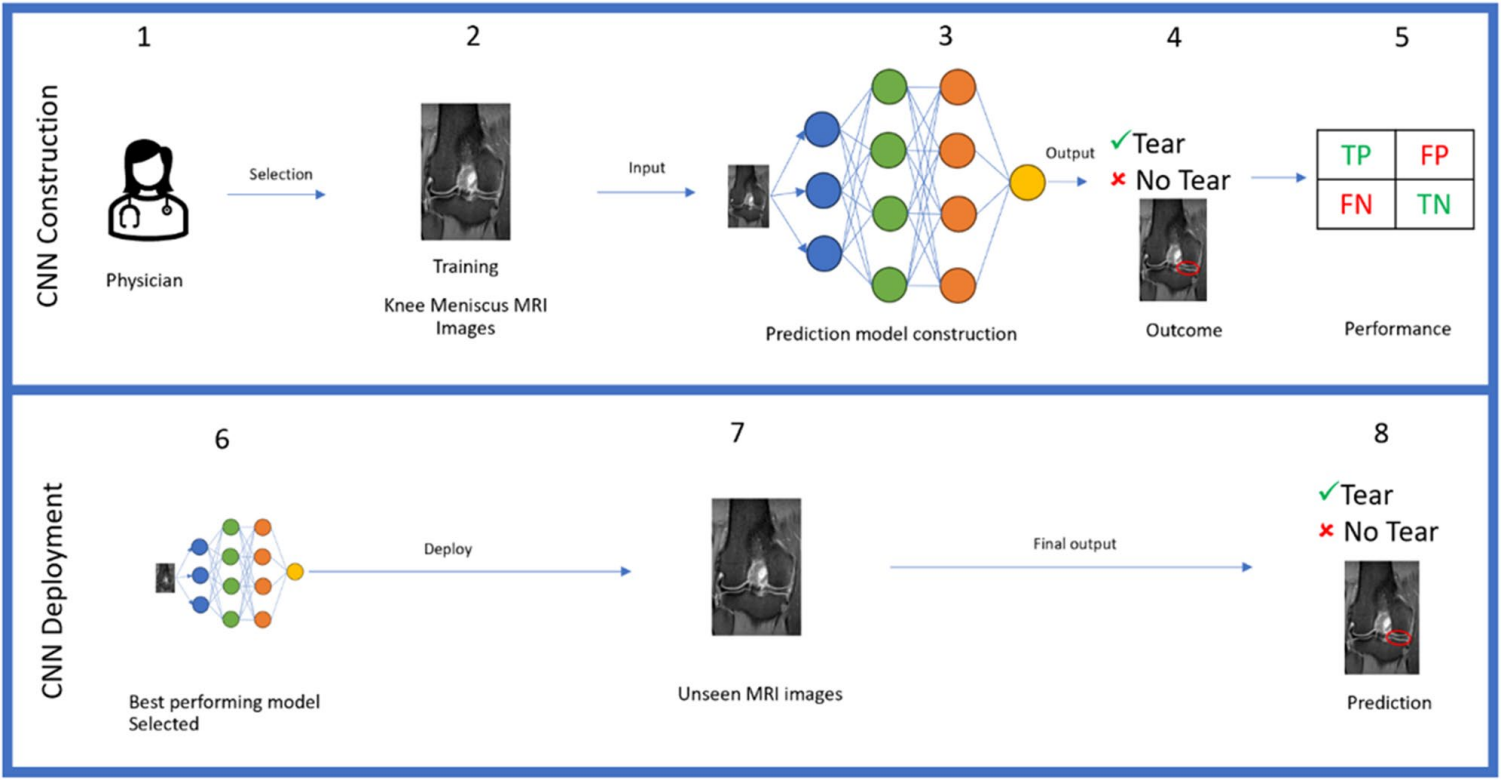
Overview of deep learning convolutional neural network. The development of a CNN for meniscus tear prediction is split into two stages (construction and deployment). During the construction process, physician will first select the appropriate knee MRI images with and without meniscus tear (**1**,**2**) to be inputted for model construction (**3**). Here, one image is dissembled with different features represented in circles and arranged in layers for the decision-making process (**3**). Then, after the computation of the inputted information, a final decision on the MRI images is reported (**4**). This can be done quantitatively, such as “tear”/ “no tear” or qualitatively in terms of areas of interest as indicated by the red circles on the MRI image (**4**). The true positives (TP), false negatives (FN), true negatives (TN), and false positives (FP) values will be extracted, and the corresponding sensitivity and specificity values will be calculated. For the deployment stage, the best-performing model selected (**6**) will be used to process a database of unseen MRI images (**7**) and produce the final predictions of meniscus tear diagnosis (**8**). The knee MRI image used is from the author’s image collection

Convolutional neural networks (CNN), a type of AI method, work to develop predictive outcomes based on pattern recognition from inputting large volumes of complex data, such as MRI images [8, 9]. The structure of the decision-making process is based on the connection between different “layers” of variables from the inputted data as inspired by the human brain’s neural networks (Fig. 1) [9]. Each CNN “layer” extracts features from input MRI images to learn the complex relationships within the image, ultimately providing a report of the image. Once sufficiently trained, the AI may process unseen imaging studies with high accuracy and fast speeds [2, 8, 10].

For meniscal tear diagnosis, multiple studies have investigated using CNNs to detect meniscal tears [11, 12].

However, there are currently no meta-analyses comparing and summarizing all of these. While CNNs have shown high accuracy and concordance with expert clinicians, the explainability and generalizability of these models are limited by the “black box” problem—where the decision-making processes cannot be adequately explained given the complexity of its computational steps [13, 14]. This is especially critical when using CNN models within clinical settings, given the associated ethical considerations in patient care [15, 16].

## Aims

This study aimed to perform a systematic review and meta-analysis to assess the feasibility and accuracy of CNN in diagnosing meniscal tears. We also aimed to analyze the decision-making algorithms reported in these studies.

## Methods

The protocol published for this systematic review and meta-analysis was constructed according to the Preferred Reporting Items for Systematic Review and Meta-analysis Protocols (PRISMA-P) statement [17]. The review was registered on PROSPERO (https://www.crd.york.ac.uk/prospero/) under number CRD42021291219.

### Eligibility criteria

Studies investigating the use of CNN in the diagnosis of meniscus tears using knee MRI scans were included in this review. The reported performance of the models needed to include accuracy, sensitivity, specificity, and ROC values. Where the performance matrix of an included study was absent, the authors of these studies were contacted to retrieve relevant data.

### Search strategy and study selection

A systematic search was performed using the MEDLINE, PubMed, EMBASE, and Cochrane databases. The search included Medical Subject Heading (MESH) terms and free text with appropriate Boolean operators. The following terms were included in the search: “knee,” “meniscus tear,” and “diagnosis,” as well as multiple synonyms for the term “artificial intelligence” and “MRI” to account for differences in terminology. All eligible studies published between July 1977 and Dec 2022 were uploaded to Covidence [18]. A further manual search of references in all included articles was performed to identify any missed studies or additional data. The full search strategies were included in the supplementary file.

### Data collection and outcome measures

**Fig. 2:**
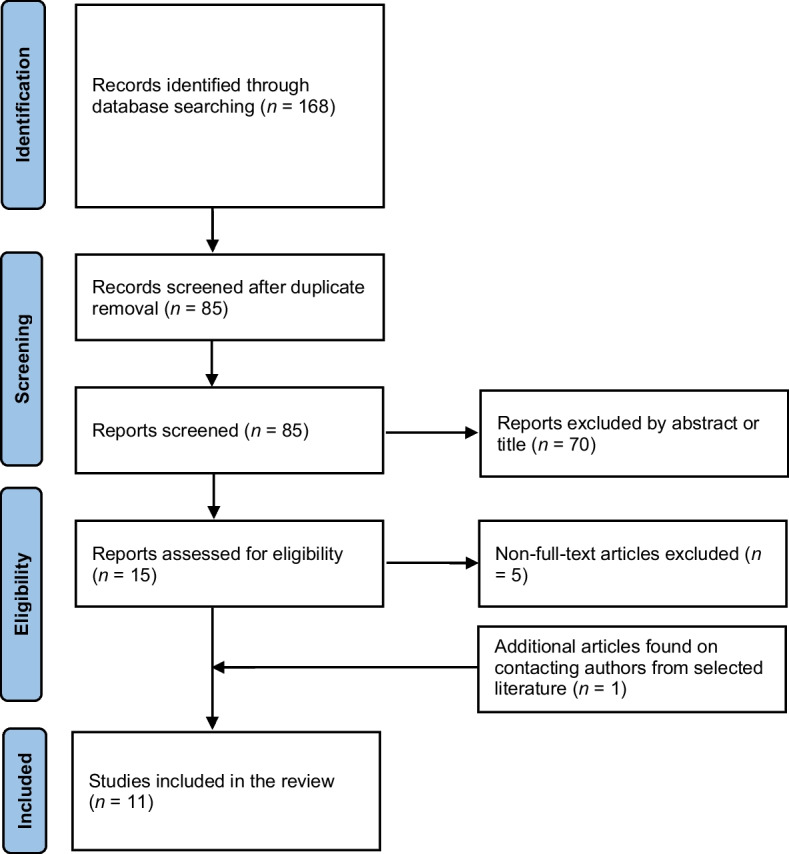
PRISMA flow diagram of evidence acquisition. PRISMA = Preferred Reporting Items for Systematic Reviews and Meta-analysis

Two reviewers screened eligible studies independently by assessing titles and abstracts. The full text was retrieved for further review if an article was considered eligible. Disagreements between reviewers were discussed to reach a consensus or consult a third reviewer. Excluded articles were noted for further analysis, and the reasons were documented in detail to generate the PRISMA flow diagram (Fig. 2). Studies that did not investigate the diagnosis of meniscus tears on MRI imaging, such as those investigating ligament damage, were excluded. Studies that did not focus on the knee or did not use MRI imaging were also excluded. Correspondence articles, expert opinions, conference abstracts, review articles, and case reports were excluded. Selected studies needed to be written in the English language.

The primary endpoint was statistically significant differences in quantitative measurements (sensitivity, specificity, and ROC), determining the diagnostic accuracy of different AI models. In addition, we focused on key themes within the literature, such as the MRI scoring system and the criteria used to define a meniscus tear.

### Quality and bias assessment

The QUADAS-2 tool was used to assess the bias of each included study [19]. The scoring system is split into four main sections: participants, predictors, outcome, and analysis. Within each section, signaling questions assessed the quality of the research methodology and results at the study level. Two reviewers were independently involved in this process, with disagreements settled by consensus. The outcome of the bias assessment was used to influence data synthesis by assessing the applicability and reliability of the data produced. As this review was aimed at the diagnostic accuracies of ML models within knee MRIs, any non-applicable sections of the scoring systems were altered to reflect the nature of the evidence base and reduce reporting errors.

### Statistical analysis / meta-analysis

The pooled quantitative diagnostic accuracy values for the AI models were compared in the meta-analysis. In the first instance, sensitivity and specificity values were retrieved or calculated. If studies did not provide these values, they were calculated from clinical tables or requested by authors. When a substantial proportion of articles used other metrics, these values were retrieved and calculated separately.

The Cochrane guideline was consulted for inter-study variation, and heterogeneity was quantified through *I*^2^ [20]. As the true effect size for all studies was unlikely to be identical (due to methodological differences, institutional differences, and interpretation differences), a random-effects model was fitted for estimation with partial pooling. Subgroup analyses were conducted to explore the sources of heterogeneity. Once heterogeneity was minimized and outliers removed, summary estimates were given for the prediction accuracy of AI models. All data analysis and visualization were performed via the R statistical environment (version 4.2.2, 2022–10-31) using the “mada” and “mvmeta” packages, Revman v5.0, and MetaDTA, an online tool for diagnostic accuracy of meta-analyses [21].

## Results

### Literature identification

The database search yielded 168 results (Fig. 2). A total of 15 articles were included in the full-text analysis after removing duplicates and assessing titles and abstracts. Five studies were removed due to non-diagnostic CNN applications. Of the 11 resultant studies, one study was included in the meta-analysis after author contacts for the relevant data [22]. Three studies were excluded from the meta-analysis as the full data could not be obtained but included in the systematic review [12, 23, 24].

### Study characteristics

Tables 1 and 2 illustrate the study characteristics. The full extracted data of included studies is included in Table S1 (supplement). Eleven studies were included in the systematic review and meta-analysis. Included studies were published from 2016 to 2022. A total of 13,467 patients and 57,551 images were included. Two studies did not specify the number of patients included [23, 24].

**Table 1:** Characteristics of the included studies

Study characteristics	No. of studies (*n* = 11)
Imaging plane	
Coronal	4
Sagittal	11
Transverse	3
Scan sequence	
3D FAST SPIN ECHO	2
3D DESS	2
DIXON	1
T1	3
T2	1
T2 FS	2
PD	1
PD FS	5
IW	1
IW FS	2
STIR	1
Scan thickness	
< 1 mm	2
> 1 mm	5
Not specified	4
Outcome classification	
Presence of lesion	8
Location of lesion	3
Deep learning methods	
Convolutional neural network	10
Perceptron neural network	1
Pre-training	
Yes	5
No	6
Reference standard	
Radiologist	4
Surgery	1
Others	6
Inter-observer agreement	
Yes	4
No	7
External validation	
Yes	4
No	7
Augmentation	
Bounding box	8
Classification mapping	2
Not specified	1

**Table 2:** Descriptive statistics of development and external validation datasets in the included studies

Study characteristics	Development datasets	External validation datasets
Number of images		
Median	1823	180
Interquartile range	1403–5259	115–440
Range	248–20,520	50–700
Percentage of “positive” diagnosis (%)		
Median	30%	**-**
Interquartile range	14–43%	**-**
Range	12–75%	**-**

### CNN methodologies

All studies included sagittal knee MRI images, four studies included coronal images [11, 25–27], two studies included transverse images [26, 28], and one study included axial images [11]. Proton density with fat-suppression sequence was the most common scan sequence used. Outcome class proportion of *no tear: tear* of the input dataset ranged from 1:0.13 to 1:3.00 [25, 29].

All studies used CNNs in different variations (Table S1). Three studies used three-dimensional CNNs where the input images were two or more dimensions [25, 27, 30]. Region-based CNNs were used in three studies where the input data was labeled in bounding boxes [22–24]. One study investigated the Perception Neural Network, an alternative form of a CNN [29]. Fritz et al utilized a Deep-CNN where the model had more processing layers for the diagnostic outcome prediction [26].

Five studies incorporated model pre-training [12, 22, 24, 25, 30]. Regarding the reference standard for identifying meniscus tears on MRI scans, eight studies engaged qualified radiologists to diagnose meniscal tears on MRI [11, 12, 22, 23, 25, 28–30]. One study extracted the diagnostic conclusion using natural language processing algorithms of the MRI reports but did not consult directly with a clinician [27], one study utilized knee arthroscopy as the reference standard [26], and one study obtained their images already marked by a database [24].

### Model validation

Four studies reported inter-observer agreement for labeling the data [25–27, 30]. Eight studies conducted internal validation, and four studies conducted external validation [11, 12, 22, 24–27, 29, 30]. The image augmentation to demonstrate the lesions involved bounding boxes and heat maps, used in eight [12, 22–25, 27, 29, 30] and two papers [11, 26], respectively. One article did not specify the method of image augmentation [28].

### Meta-analysis

The validated results for each AI model were used for the meta-analysis. Three studies conducted external validations [11, 22, 27], and six studies conducted internal validations [22, 25, 26, 28–30]. The area under the curve of the receiver operating curve (AUC), sensitivity, and specificity for diagnosing meniscus tears were reported separately in tear identification and tear location levels for the meta-analysis. Multi-class outcomes were converted into binary outcomes. Three studies were excluded from the meta-analysis due to incomplete data [12, 23, 24].

Articles in the tear identification analysis identified the presence of a meniscal lesion in an overall MRI of the knee. In contrast, articles in the tear location analysis divided each meniscus into four horns (medial, lateral, anterior, and posterior) and reported the diagnostic performance of the AI model in individual horns.

### Tear identification analysis

**Fig. 3:**
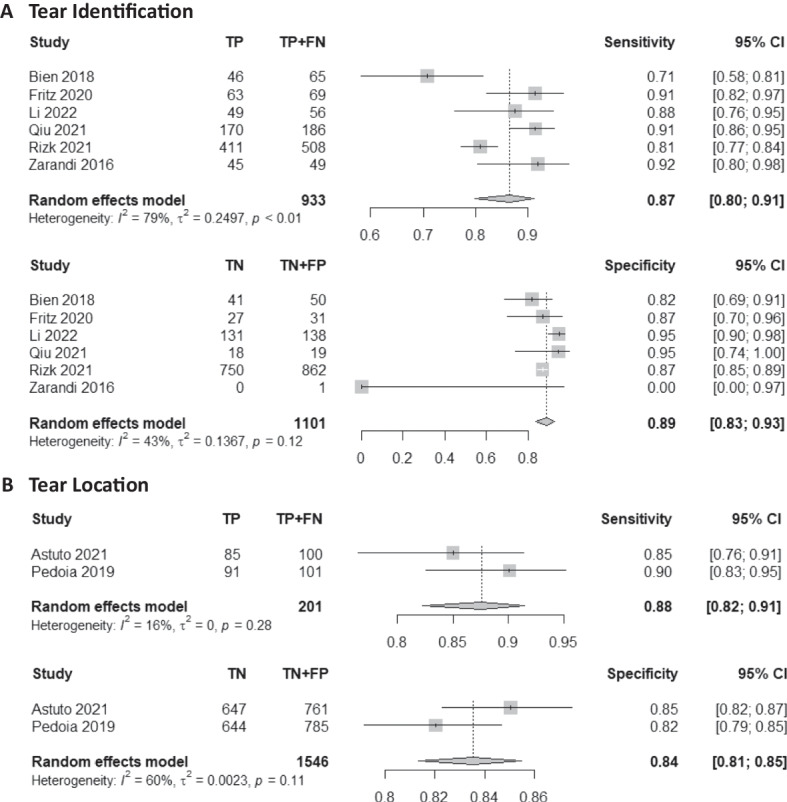
Forest plot representing the reported sensitivity and specificity values for tear identification analysis (**A**) and tear location analysis (**B**). The sensitivities and specificities with a 95% confidence interval of individual studies were indicated by squares and lines extending from their center. The pooled sensitivities and specificities were displayed in bold and as diamonds in the graphs

**Fig. 4:**
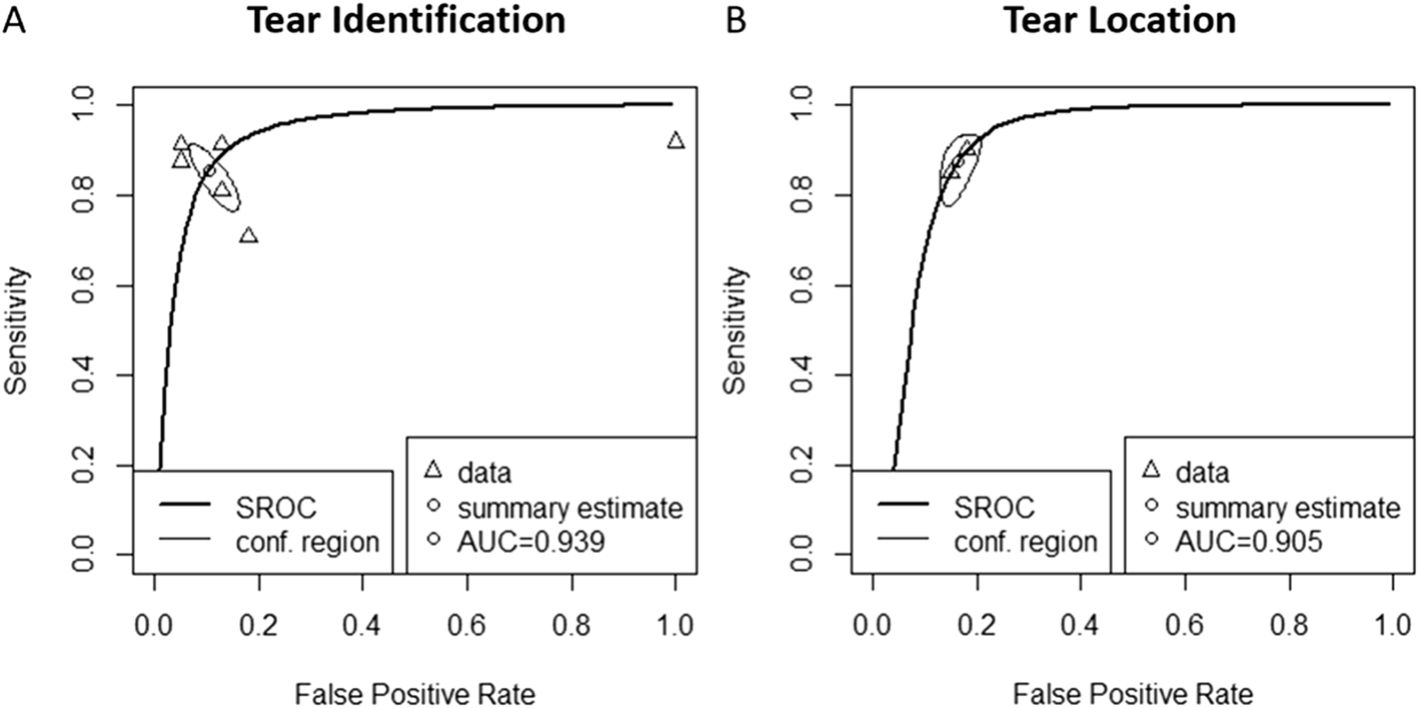
Receiver operating curves (ROC) for tear identification analysis (**A**) and tear location analysis (**B**). The SROC curve indicates the summary estimate in a circle. Triangles represent the included study, with dotted lines representing the confidence interval and solid lines for the SROCs. AUC values are displayed in the legend. SROC = summary receiver operating characteristic

Six studies were included in the tear identification analysis of meniscal tears [11, 22, 26–29]. A total of 2034 images were included, with 933 images proven to have a meniscus tear. The pooled sensitivity for the use of AI models was 0.87 (95% CI 0.80–0.91), and the pooled specificity was 0.89 (95% CI 0.83–0.93, Fig. 3), with the AUC value of 0.939 (Fig. 4). The heterogeneity was large for the sensitivity analysis (*I*^2^ = 79%) with a statically significant Cochrane Q statistic *p* < 0.01 but was insignificant for the specificity analysis (*p* = 0.12).

### Tear location analysis

Two studies were included in the tear location analysis [25, 30]. In total, 1747 meniscus horns were included, with 201 torn horns. The pooled sensitivity for the use of AI models was 0.88 (95% CI 0.82–0.91), and the pooled specificity was 0.84 (95% CI 0.81–0.85, Fig. 3), with the AUC value of 0.905 (Fig. 4). The heterogeneity was low (*I*^2^ = 16%) for the sensitivity analysis but was moderate (*I*^2^ = 60%) for the specificity analysis with statically insignificant Cochrane Q statistics (*p* = 0.28 and *p* = 0.11). One study was not included in this subgroup analysis due to the unavailability of the complete data [12].

### Risk of bias and study quality

**Fig. 5:**
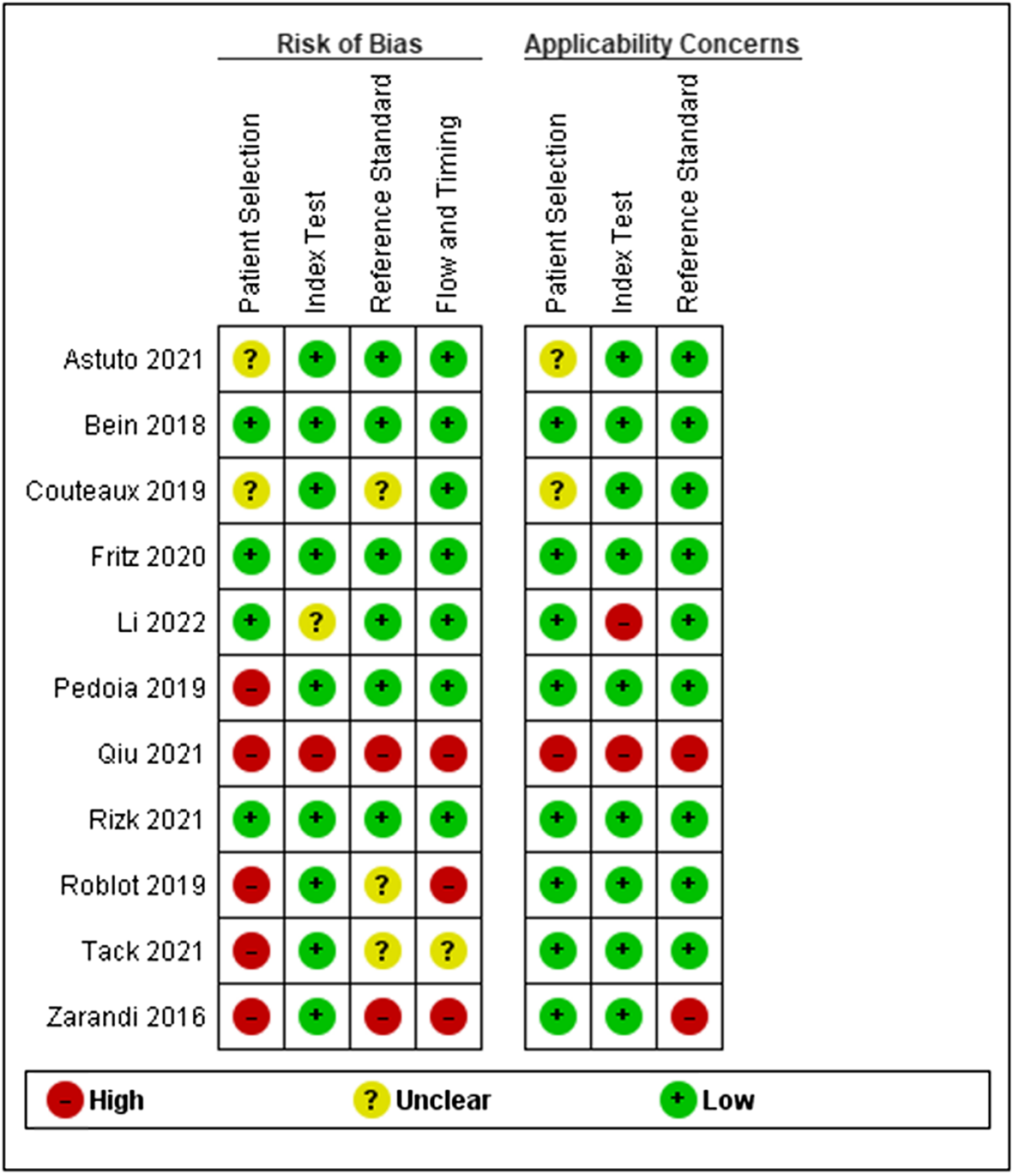
QUADAS-2 score indicating the risk of bias analysis in assessing the low, high, or unclear risk for patient selection, index test, reference standard, flow, and timing for individual included studies. An add-on analysis on applicability concerns is also included

The QUDAS-2 tool was used for quality assessment [19]. Figure 5 illustrates the summary risk of bias for the included studies. There was a high risk of bias (ROB) for patient selection within the articles. Only two studies reported clear exclusion criteria [25, 27]. Roblot et al studied external data from a dataset challenge and failed to describe excluded patients [23]. The index test was reported well with clear descriptions of algorithm development, resulting in a low ROB score in 90% of studies. Qiu et al scored poorly in this domain as they were unable to demonstrate a clear methodology and also required both MRI and CT images in their testing, reducing the clinical applicability [28]. The reference standard used among the studies varied from using several specialist radiologists and arthroscopy-confirmed injuries to no reported diagnostic standard.

**Fig. 6:**
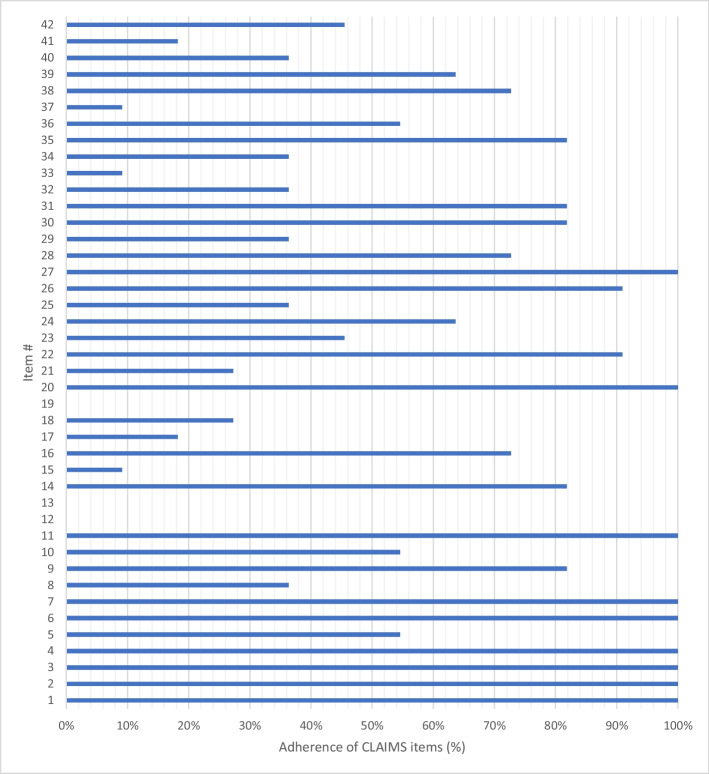
The level of adherence to the CLAIM checklist in the form of item-by-item analysis

The CLAIM-AI checklist was used to assess the specific adherence to the reporting guidelines of the included study [31]. Figure 6 illustrates the level of adherence to individual items listed in the checklist from the included studies. Items 12, “*Describe the methods by which data have been de-identified and how protected health information has been removed*”; 13, “*State clearly how missing data were handled, such as replacing them with approximate or predicted values”;* and 19, *“Describe the sample size and how it was determined”* were not followed by any of the included articles.

## Discussion

We performed a systematic review and meta-analysis to assess the accuracy of CNN in the diagnosis of meniscus tears. The CNN algorithms accurately identified the presence of tears (AUC = 0.939) but less accurately identified the location of these tears (AUC = 0.905).

None of the included studies deployed a reporting standard, such as CLAIM-AI, in the methodology, which may contribute to the heterogeneity observed in both meta-analyses [31]. This is crucial in evaluating AI studies as they encourage full reporting of the study data and algorithm methods and indicate a robust clinical scope of the algorithm output [15, 32]. Existing study lacked an explanation regarding methods of anonymization, rationale behind choosing the number of participants required for the construction of the model, and methods of handling missing data.

The current performance reporting matrix, such as the AUC alone, may not be sufficient in fully reporting the performance of an algorithm. Halligan et al highlighted the shortfall of using AUC, including the inability to differentiate the prevalence of false negatives and false positives, thereby being unable to depict the cost associated with both false predictions in AI studies [33]. Moreover, AUC may be falsely elevated with the presence of imbalanced data, meaning the proportion of each class does not equate to each other; therefore, it may not fully report the true detection performance [34]. In our study, eight studies included a higher proportion of negative class (no tear) than positive class (tear) [11, 12, 22–25, 27, 28, 30]. Data imbalance remains a challenge in the methodology of AI studies as the real-world disease prevalence is usually low for meniscus tears [35–38]. Therefore, studies may consider reducing the training data size to ensure an equal proportion of outcome classes and incorporate other AI-specific performance matrices such as precision, recall, F1 score, and F1-precision curve to better inform the shortfalls of the predictive outcomes [15, 34]. Moreover, Namdar and colleagues proposed a modified AUC for neural networks that future studies may consider as it considers algorithm prediction’s confidence [39].

MRI remained the gold standard for meniscus tear diagnosis, yet the inter- and intra-observer variability may impact the diagnostic accuracy of the MRI images [40, 41]. In our review, four studies incorporated imaging scoring to aid the classification of meniscus tears [12, 25, 28, 30], while Six studies did not specify the decision-making process of identifying meniscus tears [11, 23, 24, 26, 27, 29]. Astuto et al demonstrated that experience might impact the diagnosis of a meniscus tear in reading the MRI images with inter-reader agreement values ranging between 0.46 and 0.57 and between attending radiologists and trainees [25]. However, the AI algorithm improved the agreement score to 0.67–0.70 [25]. This demonstrated the feasibility of incorporating the prediction model in the diagnosis process. Moreover, Bien et al reported a moderate inter-observer score (0.745) for detecting meniscus tears but did not investigate the impact of AI algorithms [11].

### Future implications

To confirm the clinical utility of the prediction models, future studies may investigate the improvement of radiologist performance with deep learning. Astuto and colleagues demonstrated the improvement of intergrader Cohen ĸ agreement with CNN-assisted diagnosis [25]. There may also be a role for AI-assisted diagnosis of meniscus tears when a disagreement exists between reporting radiologists or a missed tear that has been retrospectively diagnosed [42]. However, our results suggested that the presence of tears is more accurately diagnosed than the location of tears.

Furthermore, the ability of CNNs to incorporate clinical data beyond imaging data may be useful in investigating the impact of meniscus tears on patient outcomes. The prediction outcomes could extend beyond merely the presence of a meniscus tear to the function, progression, and need for treatment for individual patients [43, 44]. Natural language processing (NLP) models may become widely adapted in future studies to generate a large dataset for model training, as conducted by Rizk and colleagues [27, 45]. This may be delivered by incorporating NLP in future research to identify images needed for a particular research focus [46].

Differentiating the location of meniscus tears through CNN methods may better inform the management of meniscus tears, given the varying disease process of the tears [47, 48]. Although not investigated in the current studies, classifying the pattern of meniscus tears by the anatomical vascular zones of the meniscus may aid the management plan, given its varying healing properties [48–50]. Future studies may focus on the pathological characteristics of tears to strengthen the clinical utilities of the prediction results, such as assessing the likely outcomes of surgical interventions.

### Limitations

There are several limitations to the study. Firstly, the heterogeneity in the meta-analysis should be interpreted with the methods of the included studies. There was significant heterogenicity in the image sequences obtained and used to train and evaluate each model. While there is no defined standard for MRI knee evaluation, only two studies used a “typical” 3-plane acquisition to develop its models, and one article did not document which sequences were used. This significantly limits the application of any algorithm to a real-world environment. Secondly, although this study attempted to separate different diagnosis outcomes of the meniscus tear, the number of included studies in individual analysis remained small. This, therefore, may not fully explore the overall performance of AI models in these subgroups. Thirdly, none of the included studies abides by AI-specific standardized reporting guidelines, which may limit the applicability of the results in clinical practice.

## Conclusion

Our study suggests that CNN is accurate in confirming meniscus tears, but there is room for improvement when assessing the location of the tears. The clinical utilities of the predictive outcomes should be continually assessed through standardized reporting, external validation, and full reports of their performances. Reporting CNN-assisted diagnostic performance may assess its function in clinical practice. Future studies are necessary to investigate the implication of different patterns of meniscus tears to determine the management plan in individual patients.

## Supplementary Information

Below is the link to the electronic supplementary material.

Supplementary file1 (PDF 146 KB)

## Abbreviations

CI: Confidence interval
CNN: Convolutional neural networks
NLP: Natural language processing
ROB: Risk of bias

## Declarations

### Guarantor

The scientific guarantor of this publication is Yi Zhao.

### Statistics and biometry

No complex statistical methods were necessary for this paper.

### Methodology

• retrospective

• diagnostic study

• multicenter study
