# Correction: Artificial intelligence applied to magnetic resonance imaging reliably detects the presence, but not the location, of meniscus tears: a systematic review and meta-analysis

**DOI:** 10.1007/s00330-024-11154-z

**Published:** 2024-10-29

**Authors:** Yi Zhao, Andrew Coppola, Urvi Karamchandani, Dimitri Amiras, Chinmay M. Gupte

**Affiliations:** 1 Imperial College London School of Medicine, Exhibition Rd, South Kensington, London, SW7 2BU UK; 2 Imperial College London NHS Trust, London, UK

**Correction to: European Radiology**

10.1007/s00330-024-10625-7, published online 22 February 2024

In Figure 2 of this article, in the text box “Reports excluded by abstract or title”, the number “68” should have been “70”.

The correct Fig. 2 is as follows:
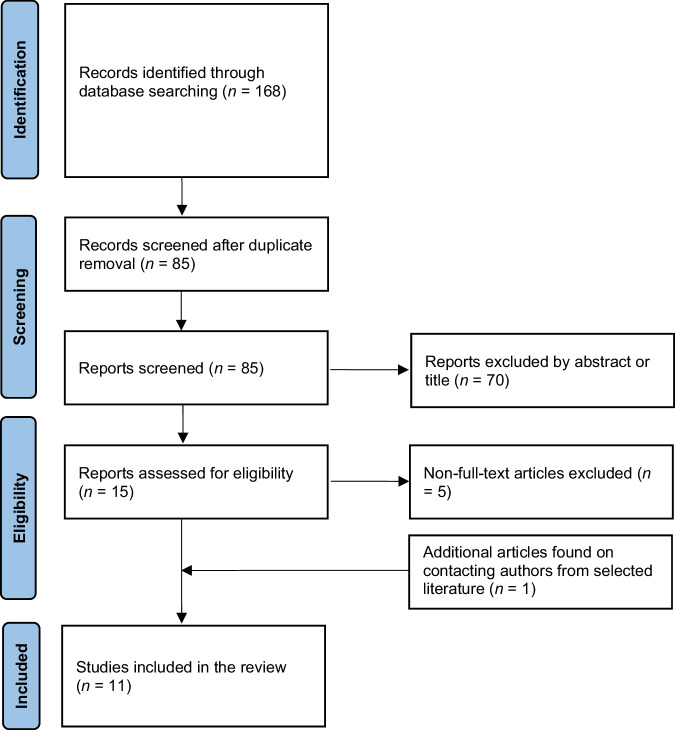


**Fig. 2** PRISMA flow diagram of evidence acquisition. PRISMA, Preferred Reporting Items for Systematic Reviews and Meta-analysis

The original article has been corrected.

