# Supplementary material for: Artificial intelligence applied to magnetic resonance imaging reliably detects the presence, but not the location, of meniscus tears: a systematic review and meta-analysis
Source: Eur Radiol. 2024 Feb 22;34(9):5954–64. doi: 10.1007/s00330-024-10625-7 (PMC11364796; doi:10.1007/s00330-024-10625-7)
Supplement: Supplementary file 1 — Supplementary file1 (PDF 146 KB) [file 330_2024_10625_MOESM1_ESM.pdf]

Artificial intelligence applied to magnetic resonance imaging reliably detects the presence, but not the location of, meniscus tears: a systematic review and meta-analysis

Electronic Supplementary Material (ESM)

Eur Radiol (2024) Zhao Y, Coppola A, Karamchandani U, Amiras D, Gupte CM

Study Characteristics	No. of patients	No. of Images	Imaging plane	Scan sequence	Scanning thickness	Meniscus segments	Outcome class proportion in training dataset: No tear/tear (ratio)	CNN Method	Pre-training	Reference standard	Is inter-observer agreement included?	External validation	Augmentation
Astuto 2021 (25)	294	1435 (5740 horns)	Coronal; Sagittal	3D-FAST SPIN ECHO	0.5mm	MA; MP; LA; LP	5073: 667 (1:0.13)	3D-CNN	V-Net	Five board-Certified Radiologist	Yes	50	Bounding Box
Qiu 2021 (28)	205	2,460	Sagittal,	Sagittal T1; Sagittal T2	3mm	Meniscus injury	128: 77 (1:0.60)	CNN	NA	One professional Doctor	NA	NA	NA
Zarandi 2016 (29)	28	248	Sagittal	PD	NA	Medial; Lateral	7:21 patients (1: 3.00)	Perceptron Neural Network	NA	Clinical expert	NA	NA	Bounding box
Bien 2018 (11)	1312	1370 exams	Sagittal; Coronal; Axial	Coronal T2 FS, Coronal T1, Sagittal PD FS Sagittal T2 FS Axial PD FS	2.5-4mm	Sagittal; Coronal; Axial	921: 449 (1:0.49)	CNN	NA	Three radiologists	NA	NA	Class activation mappings
Tack 2021 (12)	2399	2,399	Sagittal	3D DESS; Sagittal IW FS	0.7-3.0mm	MA; MP; LA; LP	NA	CNN	ResNet50; Dilation ResNet-C-26	Clinical experts	NA	NA	Bounding box
Pedoia 2019 (30)	302	1478 studies (5912 horns)	Sagittal	3D-FAST SPIN ECHO	0.5mm	MA; MP; LA; LP	1039:160 (1:0.15)	3D-CNN	2D U-net	Five board-Certified Radiologist	Yes	NA	Bounding Box
Couteaux 2019 (24)	Not specified	1128	Sagittal	NA	NA	Whole meniscus; Horizontal; Horizontal posterior; Horizonal anterior; Vertical posterior; Vertical anterior	828:300 (1:0.36)	R-CNN	ResNet-101	Images provided by the French Society of Radiology	NA	NA	Bounding Box
Fritz 2020 (26)	100	20,520 MRI Studies	Coronal, Transverse, Sagittal	Coronal T1 Coronal STIR Axial IW FS Sagittal IW DIXON	2.5-3mm	Medial; Lateral	31:69 patients (1: 2.23)	DCNN	NA	Arthroscopic knee surgery	Yes	NA	Heat map
Roblot 2019 (23)	Not specified	1823	Sagittal	T2	NA	Anterior; Posterior	1948/298 (1:0.15)	R-CNN	NA	Direct labeling by authors	NA	700	Bounding Box
Rizk 2021 (27)	7903	8058 MRI examinations	Coronal; Sagittal	FS PD	NA	Medial; Lateral	4306/3453 (1:0.80)	3D-CNN	NA	Reports were extracted using Natural Language Processing algorithms	Yes	MRNet database	Bounding Box
Li 2022 (22)	924	16632 (augmented 20x by labeling)	Sagittal	FS PD	3mm	Anterior; Posterior; Meniscus body	25060/5020 (1:0.20)	R–CNN	ResNet50	A board-certified radiologist and a board-certified sports medicine physician.	NA	180	Bounding box and mask diagnosis

Table S1: Descriptive Statistics for the included studies. 3D = Three dimension; 3D-CNN = Three-dimensional convolutional neural network; CNN = Convolutional neural network; DCNN = Deep convolutional neural network; DESS = Double Echo Steady-State; FS = Fat-suppressed; FSE = Fast spin-echo; IW= intermediate weighted; LA = Lateral Anterior; LP = Lateral Posterior; MA = Medial Anterior; MP=Medial Posterior; NA = Not applicable ; PD = Proton density weighed; R-CNN = Region-based convolutional neural network; STIR = short-tau inversion recovery; T1 = T1-weighed images; T2 = T2-weighed images

Full Search Strategy

(artificial intelligence OR neural network OR CNN OR deep learning OR machine learning OR machine intelligence) AND (menisc* OR cartilage tear OR flap tear OR torn meniscus OR root tear OR bucket handle tear OR radial tear OR cleavage tear OR longitudinal tear OR parrot beak tear OR horizontal tear) AND (MRI OR MR OR magnetic resonance imaging OR MP-MRI OR multi-parametric MRI OR multi-parametric magnetic resonance imaging OR Imaging OR multiparametric MRI OR "multiparametric magnetic resonance imaging")*
